# Around the Hospitals

**Published:** 1894-06-30

**Authors:** 


### Around the Hospitals.

[Notice to the Managers of Institutions.—Special space is now reserved for the insertion of notices of hospital meetings and festivals. The Editor requests that all notices may reach The Hospital Office, 428, Strand, at least one week before the date of each meeting.]

**The Hospital for Sick Children, Great Ormond Street.**—The chief event of the past year at this institution was the opening of the new wing by the Prince and Princess of Wales. The Princess showed her interest and sympathy with the little sufferers by presenting each child personally with a toy. The enlargement of the hospital was much called for, but will, of course, entail extra expenditure. At one time the Hospital for Sick Children was perhaps the most popular hospital with the public throughout London. It must be admitted that interest from without has not been as keen for some time as formerly. Every effort, nevertheless, is necessary to stimulate public support, as the needs of the institution are very urgent. In order to meet expenses, contributions through legacies have to be expended, instead of their being placed to capital account. During 1893 two new cots were endowed—one by Mrs. Bridge, and one by the Whitfield Tabernacle Sunday-school. The in-patients treated amounted to 1,604, and out-patients to 27,698, both showing a considerable increase over the numbers of the previous year. The total expenditure amounted to £14,975. At the convalescent branch 278 children were admitted.

**The Dental Hospital, Leicester Square.**—Strenuous efforts are being made by those interested in securing sufficient funds to defray the cost of the new buildings held to be necessary for the satisfactory working of the hospital. Property for the site has already been purchased, for which purpose a loan of £15,000 has been raised at the bankers, and this the committee are most anxious to clear off. Amongst the developments in the work of the hospital may be mentioned the department for correcting irregularities in children's teeth, a most useful and beneficial undertaking. The hospital is now open in the afternoon as well as the morning. During the year 55,325 cases were treated.

**The Westminster Hospital.**—Beyond some important changes in the medical and surgical staff, the year 1893 was not marked at the Westminster Hospital by any particular event. More work was done, and as has been generally the case, less income was forthcoming. The authorities are strenuous in their efforts to meet the calls upon them, and consequently some of the capital has to be realised to meet the necessary expenditure. This expenditure in 1893 amounted to £13,898. The expenses are carefully regulated, and the past year shows a lessened cost per head per patient over that of 1891, which must be taken as a parallel year owing to the cleaning and painting of the institution being effected on alternative years, 1893 being one of these. The cost per in-patient last year amounted to £71 2s. 6d. These patients numbered 2,950, whilst out-patients reached a total of 26,396. The convalescent branch of the hospital's work is a most useful one. Fifteen beds at the Parkwood Convalescent Home are allotted to the use of the Westminster Hospital, and prove of immense service.

